# Drug hypersensitivity in children in Brazil

**DOI:** 10.1186/2045-7022-4-S3-P145

**Published:** 2014-07-18

**Authors:** Luis Felipe Ensina, Ines Cristina Camelo-Nunes, Mara Morelo Felix, Maria Fernanda Malaman, Gladys Queiroz, Djanira Martins Andrade, Ligia de Oliveira Machado, Alex Eustaquio Lacerda, Camila Teles Machado Pereira, Dirceu Sole

**Affiliations:** 1 Federal University of Sao Paulo, Allergy, Immunology and Rheumatology, Brazil; 2 Hospital Federal dos Servidores do Estado do Rio de Janeiro, Allergy and Immunology, Brazil; 3 Universidade Tiradentes, Allergy and Immunology, Brazil; 4 Federal University of Pernambuco, Pediatrics, Brazil

## Background

Drug hypersensitivity is one of the most frequent reasons for consultations with an allergist in Brazil. However, drug hypersensitivity epidemiology data in children is scarce. The aim of this study was to investigate children reporting suspected drug hypersensitivity reaction (DHR).

## Methods

From June 2011 to December 2013 a prospective observational study was implemented in 4 allergology units from different regions of Brazil. Children reporting DHR were evaluated using a modified ENDA questionnaire, and a standardized diagnostic work up was performed.

## Results

Ninety patients were evaluated, 54 male, with a median age of 6 years. Personal history of atopy was reported in 65 and previous DHR in 9. Cutaneous manifestations were observed in 86 -- urticaria and/or angioedema in 71 and macular or maculopapular exanthema in 15. Other symptoms reported were: respiratory (25), gastrointestinal (8), cardiovascular (5). The interval between dose and reaction was less than 1 hour in 38 subjects. Mild reaction was observed in 32 patients and moderate in 55. Fever and/or viral infection were present in 61 patients during or just before the reaction. The majority of subjects were treated in emergency units (79). More than one drug was suspected as a trigger in 50 children (NSAIDs in 50%, beta-lactam antibiotics in 31% and other antibiotics in 8.5%). Sixteen skin tests (prick and intradermal) were performed and were all negative but one with amoxicilin. Drug provocation tests were positive in 4 of 51 tests - NSAIDs 29 (4 positive), beta-lactam antibiotics 20 and others 4. Sixty-one reactions were possible or probable related with the suspected drug, but in 25 this relation was unlikely.

## Conclusions

Children with a suspected history must be fully investigated to confirm or exclude the diagnosis of DHR.

